# Supplementary material for: A Pilot Study on Plasma and Urine Neurotransmitter Levels in Children with Tic Disorders
Source: Brain Sci. 2022 Jul 4;12(7):880. doi: 10.3390/brainsci12070880 (PMC9313232; doi:10.3390/brainsci12070880)
Supplement: Supplementary file 1 [file brainsci-12-00880-s001.zip › brainsci-1750204-supplementary.pdf]

Table S1. Sample size and detection rate of each neurotransmitter in plasma.

	3–7-year-old	8–12-year-old
Dopamine (DA)	31 (83.78%)	52 (91.23%)
Epinephrine (E)	35 (94.59%)	57 (100%)
Norepinephrine (NE)	37 (100%)	57 (100%)
Metanephrine (MN)	5 (13.51%)	7 (12.28%)
Normetanephrine (NMN)	32 (86.49%)	51 (89.47)
3-Methoxytyramine	0 (0%)	0 (0%)
5-Hydroxytryptamine (5-HT)	37 (100%)	57 (100%)
Glutamic Acid (Glu)	37 (100%)	57 (100%)
Tyrosine (Tyr)	37 (100%)	57 (100%)
Tryptophan (Trp)	37 (100%)	57 (100%)
γ-Aminobutyric acid (γ-GABA)	35 (94.59%)	49 (85.96%)

Table S2. Sample size and detection rate of each neurotransmitter in urine.

	3–7-year-old	8–12-year-old
Dopamine (DA)	24 (100%)	45 (100%)
Epinephrine (E)	24 (100%)	45 (100%)
Norepinephrine (NE)	24 (100%)	45 (100%)
Metanephrine (MN)	24 (100%)	45 (100%)
Normetanephrine (NMN)	24 (100%)	45 (100%)
Vanillylmandelic Acid (VMA)	24 (100%)	45 (100%)
Homovanillic acid (HVA)	24 (100%)	45 (100%)
5-Hydroxyindoleacetic acid (5-HIAA)	24 (100%)	45 (100%)

Table S3. AUC and significance of the ROC analysis for the prediction of an individual's status in plasma.

	3–7-year-old		8–12-year-old	
	**AUC**	***P***	**AUC**	***P***
Dopamine (DA)	0.515	0.844	0.688	0.001
Epinephrine (E)	0.499	0.991	0.475	0.650
Norepinephrine (NE)	0.642	0.035	0.654	0.005
Normetanephrine (NMN)	0.597	0.184	0.720	0.000
5-Hydroxytryptamine (5-HT)	0.435	0.339	0.524	0.661
Glutamic Acid (Glu)	0.661	0.017	0.732	0.000
Tyrosine (Tyr)	0.552	0.440	0.405	0.080
Tryptophan (Trp)	0.528	0.677	0.258	0.000
γ-aminobutyric acid (γ-GABA)	0.715	0.002	0.788	0.000

Table S4. AUC and significance of the ROC analysis for the prediction of an individual's status in urine.

	**3–7-year-old**		**8–12-year-old**	
	**AUC**	***P***	**AUC**	***P***
Dopamine (DA)	0.503	0.967	0.518	0.768
Epinephrine (E)	0.491	0.918	0.362	0.025
Norepinephrine (NE)	0.583	0.322	0.623	0.045
Metanephrine (MN)	0.564	0.446	0.714	0.000
Normetanephrine (NMN)	0.696	0.020	0.741	0.000
Vanillylmandelic Acid (VMA)	0.627	0.132	0.446	0.379
Homovanillic acid (HVA)	0.591	0.279	0.357	0.019
5-Hydroxyindoleacetic acid (5-HIAA)	0.718	0.010	0.669	0.006
